# Guided Endodontics: A Literature Review

**DOI:** 10.3390/ijerph192113900

**Published:** 2022-10-26

**Authors:** Kateryna Kulinkovych-Levchuk, María Pilar Pecci-Lloret, Pablo Castelo-Baz, Miguel Ramón Pecci-Lloret, Ricardo E. Oñate-Sánchez

**Affiliations:** 1 Gerodontology and Special Care Dentistry Unit, Faculty of Medicine, IMIB-Arrixaca, Morales Meseguer Hospital, University of Murcia, 30008 Murcia, Spain; 2 Unit of Dental Pathology and Therapeutics II, School of Medicine and Dentistry, University of Santiago de Compostela, 15705 Santiago de Compostela, Spain

**Keywords:** guided endodontics, canal obliteration, literature review

## Abstract

The main objective of this paper is to perform an updated literature review of guided endodontics based on the available up-to-date scientific literature to identify and describe the technique, its benefits, and its limitations. Four electronic databases (PubMed, Scopus, Science Direct, and Web of Science) were used to perform a literature search from 1 January 2017 to 13 May 2022. After discarding duplicates, out of 1047 results, a total of 29 articles were eligible for review. Guided endodontics is a novel technique that is currently evolving. It is applied in multiple treatments, especially in accessing and locating root canals in teeth with pulp canal obliteration, microsurgical endodontics, and removing glass fiber posts in endodontic retreatments. In addition, it is independent of an operator’s experience, requires less treatment time for the patient, and is more accurate and safer than conventional endodontics.

## 1. Introduction

Pulp obliteration (PO) is characterized as radiographic evidence of increased dentine production, primarily in response to trauma. The result of this process is a calcified canal, which does not necessarily indicate diseased pulp. The term pulp calcification can also be used to refer to this condition. Parallelly, the term calcific metamorphosis is defined as a pulp response to trauma characterized by the rapid deposition of hard tissue in the pulp space [1,2]. The entire pulp space may appear radiographically obliterated due to extensive mineralized tissue deposition, although some portion of the pulp space may remain in histological sections [3]. PO may be total (the pulp chamber and root canals are difficult to visualize or not visible) or partial (the pulp chamber is indistinguishable and root canals are significantly narrow but visible) [4].

Although PO is a reparative response indicating the vitality of the tooth, it can lead to pulp necrosis (PN), which is closely related to the degree of root development. One explanation for this is that the structure obstructing the pulp space contains nutrient vessels and cells that can be infected through the dentinal tubules [4]. It has also been shown that, in traumatized teeth, PN is a phenomenon that occurs in most cases [5,6].

Establishing a treatment plan for these teeth is not straightforward. Some authors have advocated prophylactic-preventive endodontic treatment of these teeth as soon as PO is diagnosed, since it is believed that the risk of PN increases after new trauma or after therapeutic treatments such as orthodontics and dental restorations [7,8]. However, at present, this guideline is not followed. Instead, it is recommended to monitor such teeth clinically and radiographically and to only perform endodontic treatment when there is clinical symptomatology or periapical tissue involvement [8].

Before, during, or after endodontic treatment of teeth exhibiting PO, a number of complications arise that can compromise both the treatment and the prognosis of the affected tooth, such as iatrogenic perforations, fractures and/or the inability to remove instrument fragments within the canals, the excessive removal of tooth tissue, or the inability to locate and negotiate heavily calcified canals. During a cavitary access in a tooth with calcified pulp, there is no asymmetric localization of the root canals nor tactile sensation of “falling into the void” after accessing the pulp chamber as in an endodontic access in a tooth without PO, so there is a high risk of perforation [8].

Magnifying glasses, microscopes, and CBCT can be used for better guidance, but it is difficult for the operator—especially a novice—to interpret the CBCT images, create a mental guide, and at the same time perform the treatment manually. Guided endodontics (GE), which is based on the use of endodontic treatment planning with the help of computer technology, emerged to solve these problems. Thus, the risk of perforations and other iatrogenic problems is reduced by creating a specific pathway for root canal access and instrumentation [9,10].

In 2016, a new approach to endodontics using 3D-printed guides or splints emerged, which was based on implant treatments that made use of the aforementioned aids to guide implant placement. Authors such as Buchgreitz J, Connert T, Krastl G, and Zhender MS, among others, published the first studies with the aim of evaluating the precision of these systems for accessing root canals, obtaining very satisfactory results [11,12]. The first case reports on the application of this technique for the treatment of teeth with OP, and traumatic antecedents also started to appear in the same year, with favorable results [13].

In addition, endodontic re-treatments can be challenging and sometimes require microsurgery. A new approach has emerged in which guided endodontics (a printed surgical guide) is used along with CBCT scans for access to the apical portion of the root during surgical endodontics. In turn, this results in more precise incisions, both in gum and bone tissues; accurate root resection; and better postoperative healing. In addition, the treatments based on this approach are less time consuming in comparison to free-hand techniques [14,15,16].

Today, there are different types of guided endodontics: static guided endodontics (SGE) and dynamic guided endodontics (DGE).

SGE is performed by obtaining a CBCT image of the patient’s upper or lower arch (depending on where the tooth to be treated is located). At the same time, a registration of the patient’s arch of interest is performed, which can be performed with an intraoral scanner or by obtaining an impression that will be scanned later. The two obtained images are superimposed through the aid of software, whereby a guide can be designed that will cover the tooth of interest (and some adjacent teeth). In this guide, a drill hole can be designed with a specific appropriate diameter and angulation to allow direct access to the calcified canal. Cylinders or “sleeves” can then be designed to allow the stable and quantified access of a drill to the interior of the root canal through the drill hole. The inner cylinder is smaller and is made of metal. Once the designs have been completed, the file is exported from the planning software in an STL (stereolithography) format for the 3D printing of the guides. If you do not have access to a 3D printer, you can send the file to a laboratory. To proceed with the use of the guides, rubber dam isolation is performed and the guide is tried on to ensure that it fits the patient’s teeth in a stable manner. The internal metal cylinder is what will guide the drill to access and remove the calcified tissue, and once it is completely removed, the root canal treatment is continued in the conventional manner [17,18].

DGE is based on the use of CBCT images with reference marks that are placed in the patient’s mouth on the side opposite to the side to be operated on (before performing the CBCT). With the help of a stereo camera connected to a dynamic navigation system, the trajectory of the drills into the pulp chamber and root canal is coordinated in real time. This way, the operator can follow everything he/she does on a monitor and can correct or adjust the angulation of the instruments as needed [19].

Guided endodontics is based on the use of endodontic treatment planning with the aid of computerized technologies. Due to the appearance of these useful techniques to cover one of the complications in endodontics, pulp obliteration, it is relevant to carry out a review of the literature to determine the advantages and/or limitations of this technique, as well as other possible applications.

## 2. Materials and Methods

A search was performed in the biomedical databases Pubmed, Science Direct, Scopus, and Web of Science for available literature from 1 January 2017 to 13 May 2022 using the following keywords and Boolean operators: “guided AND endodontics”.

Duplicates were discarded using Mendeley Desktop reference manager software (Elsevier, AMS, Asterweg, The Netherlands). Then, the titles and abstracts were manually reviewed, and those articles that did not meet the inclusion criteria were excluded. Subsequently, the remaining articles were read in full, and those that dealt with a topic other than that of interest to this literature review were excluded.

### 2.1. Inclusion Criteria

Articles were included according to the following criteria:

- Keywords: guided AND endodontics;
- Time period: last six years;
- Articles on guided endodontics: its types, uses, advantages, disadvantages, and/or outcomes of its use.

### 2.2. Exclusion Criteria

Articles were excluded according to the following criteria:

- Articles in languages other than English or Spanish;
- Animal studies;
- Reviews and systematic reviews;
- Articles that, after reading their title and abstract, did not fit in with the subject of interest of this paper.

## 3. Results

**Figure 1:**
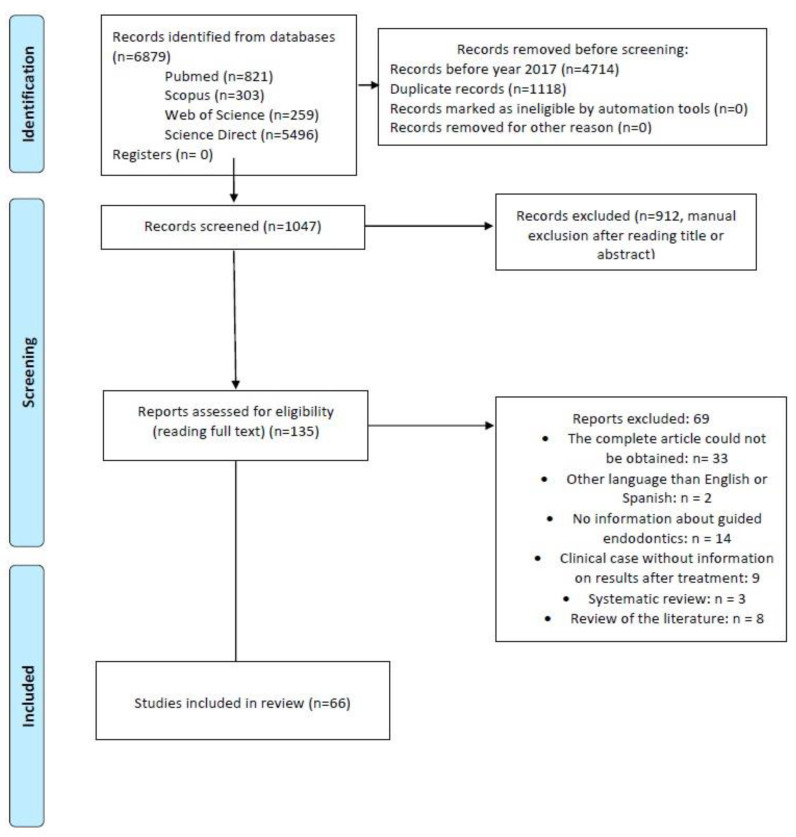
Study selection flowchart.

Once the bibliographic research was carried out, 6879 articles were obtained from the different databases (Pubmed, Science Direct, Scopus, and Web of Science), which were transferred to the Mendeley Desktop reference manager software in order to facilitate their organization and storage, as well as to discard duplicates and select only those from the last 6 years. A total of 1047 articles were obtained. Subsequently, their titles and abstracts were read, and 135 articles were eligible (excluding 912). Finally, the remaining articles were read entirely, resulting in the 66 studies that were included in this literature review (Figure 1).

### Studies Characteristics

From the 66 selected studies, the two types of guided endodontics were assessed: 53 studies analyzed SGE, and 14 studies tested DGE (Table 1 and Table 2).

From the 29 research studies, only 1 study compared SGE with DGE [20], both showing excellent results. Twelve of these studies compared GE (DGE or SGE) with manual endodontic treatment [14,15,16,20,21,22,23,24,25,26,27,28,29,30], obtaining better results. Eight of the studies were performed with 3D replicas instead of natural teeth [21,22,24,28,31,32,33,34] and only one with acrylic dentition [27] (Table 1).

The objectives of the different studies were to perform endodontic access cavity procedures, evaluate the technique with respect to the fracture strength of the teeth, guide apical access during endodontic microsurgery, perform an osteotomy and/or apicoectomy, locate the greater palatine artery to prevent its damage, remove fiberglass posts, compare 3D printers for the manufacture of SGE splints, and evaluate the results of endodontic microsurgery.

The most frequently assessed parameters from the application of SGE and DGE were effectiveness, accuracy, amount of tooth tissue removed, and speed during treatment. Table 1 provides further details on the content of these studies.

From the 37 selected case reports (Table 2), 25 of them had at least one year of follow-up with excellent results in terms of the absence of symptomatology or evidence of bone regeneration [35,36,37,38,39,40,41,42,43,44,45,46,47,48,49,50,51,52,53,54,55,56,57,58,59,60]. Eighteen of them were performed with only single-rooted teeth [3,9,38,40,44,46,50,51,52,53,55,58,60,61,62,63,64,65]. In twenty-five of them, GE was used for the treatment of pulp obliteration [3,9,35,36,37,38,39,40,41,42,43,44,45,46,47,48,49,50,51,52,54,58,60,63,64,65], eight for osteotomy and apicoectomy [57,59,61,66,67,68,69,70], two for re-treatment or removal of fiberglass posts [43,56], and one for the treatment of a dens evaginatus [55].

**Table 1:** Research articles included in review information.

Authors	Object of Study and Type of EG	Nature of Teeth and Type	Operator and Practice	Conclusions
Gambarini G et al. 2020 [21]	Ultra-conservative AC precision (DGE vs. MAN)	- R3D - type 2.6	operator with experience in both groups	DGE more precise, removes less tissue, reduces risk of iatrogenic coronary weakening
Connert T et al. 2017 [71]	SGE accuracy with miniaturized instruments	- NAT - I and C - mandibular	2 operators	SGE is accurate, fast, and operator-independent in terms of preparing apically extensive access cavities in teeth with narrow roots.
Jain SD et al. 2020 [22]	Loss of tooth tissue in AC in teeth with OP (DGE vs. MAN)	- R3D with simulated OP - 2.1 y 4.1	1 EST (with microscope for MAN access)	DGE removes less tissue and is more accurate in locating ducts with OP
Loureiro MAZ et al. 2020 [23]	Amount of tooth tissue removed in CA (SGE vs. MAN)	- NAT - I central and lateral mandibular - M (1st and 2nd) jaws	1 ESP (with magnifying glasses)	- SGE preserves more tissue in molars - No significant differences in terms of tissue removed from incisors
Connert T et al. 2021 [24]	Time and tooth loss in AC (miniaturized DGE vs. MAN)	- R3D - I (central and lateral) and C maxillae	1 operator with 12 years’ experience, 1 OP with 12 years’ experience, 1 OP with 12 years’ experience, 1 OP with 12 years’ experience.	- Miniaturized DGE results in a more precise CA and less tissue is removed. - Less experienced operators achieved comparable results to more experienced ones.
Koch GK et al. 2022 [31]	Compare 3D printers (for SGE)	- R3D - All types	1 EST	- There are significant differences between printers - All produced very accurate guides for the AC to the ducts.
Buchgreitz J et al. 2019 [72]	SGE accuracy (in teeth with OP, apical periodontitis, and in need of post)	- NAT - I (central and lateral) and C	No data	- SGE implementation is accurate in locating canals with OP in uniradicular teeth
Torres A et al. 2021 [32]	Accuracy and potential for use of DGE in AC teeth with simulated OP	- R3D - I (central and lateral), C and PM maxillary and mandibular (with OP - simulated)	1 EST, 1 ESP Yes	- EGS was accurate in performing AC on teeth with OP - The technique requires a certain degree of dexterity, manual-visual coordination - Practice is needed before treatment
Simon JC et al. 2021 [73]	Laser precision and predictability in minimally invasive CA (with DGE)	- NAT - PM and M - Subsequent	No data	- Laser integration in DGE is suitable for efficient cutting of hard dental tissues.
Su Y et al. 2021 [74]	Accuracy in AC Linear and angular deviation during AC (with SGE)	- NAT - I, C, PM and M	No data	- Acceptable accuracy of SGE during AC - Larger linear and angular deviations in M - Angular deviation best discriminates AC ability
Krug R et al. 2020 [33]	Accuracy and effort of 2 AC software (with SGE)	- R3D with simulated OP - I (central and lateral) jaws - I (central) mandibular	1 operator	- Both software packages enabled fast planning of the milling guide. - SGE treatment is predictable - Root canal localization is safe in teeth with OP
Choi Y et al. 2021 [75]	Effectiveness in CA with guidelines to prevent excessive tooth loss (student-oriented) (with SGE)	- NAT - PM and M	1 EST pre-doctoral	- Reduction in preparation time by 75.9% in PM and 81% in M when students used the AOG-3DP guide. - More conservative approaches using such guidance. - Time is needed for design and manufacture - The guide can be used for help in more difficult cases.
Ali A et al. 2021 [25]	- Effectiveness of SGE for CA through MTAs - Effect of technique on fracture strength (SGE vs. MAN)	- NAT - PM - mandibular	Same operator (with magnifiers for MAN)	- SGE results in faster and less error-prone MTA removal. - With SGE, greater resistance to tooth fracture is preserved.
Dianat O et al. 2020 [26]	Accuracy and efficiency when locating ducts with OP (DGE vs. MAN)	- NAT - I, C, PM - maxillary and mandibular with OP	1ESP AND 1 EST (2 per group)	- DGE is more accurate and efficient in locating ducts with OP - DGE can help prevent accidents during CA
Chong BS et al. 2019 [76]	Use of DGE for guided endodontics	- NAT (simulating OP) - All types	No data	- Great potential for using dynamic computerized navigation in endodontics to guide and facilitate CA and canal location.
Kostunov J et al. 2021 [27]	Success rate and tissue removal required for CA (SGE vs. MAN in teeth with OP)	- Acrylic dentition with root canals - 1.1, 1.4, 1.7	1 ESP for both groups	- The use of guided endodontics on calcified teeth allows for a considerable amount of tooth tissue to be preserved.
Jain SD et al. 2020 [34]	Minimally invasive AC and channel localisation with simulated OP (in DGE)	- R3D All types	1 ESP	- DGE together with high-speed drills achieves minimally invasive CA in canal localization with OP with an average 2D horizontal deviation of 0.9mm, 3D of 1.3mm, and 3D angular deviation of 1.7°.
Connert T et al. 2019 [28]	- Tissue loss in CA - Duration of treatment - Pipeline location and negotiation - Influence of operator experience (SGE vs. MAN in teeth with OP)	- R3D - I central maxillary - I maxillary lateral - I central mandibular	1 ESP 1 DG 1 recent graduate	SGE entails: - more predictable results - reduced loss of tooth tissue by locating calcified canals - no influence on operator experience
Zubizarreta Macho A et al. 2020 [20]	Accuracy of SGE and DGE for AC (SGE vs. DGE vs. MAN)	- NAT - I central mandibular	1 same operator for both	SGE and DGE enable more accurate CAs than conventional techniques.
Fan Y et al. 2019 [15]	Compare accuracy of OT and AP (with SGE using a grid as a guide vs. MAN)	- NAT - All types	1 ESP	The use of prefabricated grids in guided endodontic surgery proved to be more accurate than using no guide at all.
Smith BG et al. 2021 [77]	Implications of the location of the greater palatine artery in relation to the molars for the performance of OT and AP. Feasibility of a flapless palatal access technique (with SGE)	- With CBCT images of 1st and 2nd maxillary molar teeth of real patients.	2 ESP	- Endodontic (palatal root) surgery with a 2mm safety margin is possible in 47% of upper 1st molars and 52% of upper 2nd molars. A flapless palatal access can be a viable option for almost half of the maxillary 1st and 2nd molars.
Galino Buniag A et al. 2021 [78]	OT and PA results after 1 year (with SGE)	- With CBCT images of pre-treated teeth (made by the Faculty of Dental Medicine). - endodontics and residents)	2 ESP	- This study assumes that endodontic surgery using SGE has similar success rates to those using the conventional MAN technique.
Aldahmash SA et al. 2022 [29]	- Accuracy and efficiency of DGE in minimally invasive OT and AP - Feasibility of retrograde sealing - (DGE vs. MAN)	- NAT All types	1 ESP (with microscope for MAN) Yes	- DGE allowed the operator to perform minimally invasive OT, AP, and endodontic surgery as well as adequate retrograde obturation.
Dianat O et al. 2021 [16]	Accuracy and efficiency of DGE for OT and AP (DGE vs. MAN)	- NAT - All types	1 ESP (with microscope for MAN)	- DGE is more accurate than the MAN technique. - The distance of the roots from the cortical bone negatively influences the accuracy and efficiency of the MAN technique.
Gaffuri S et al. 2021 [79]	Accuracy of minimally invasive guides for OT and AP (with SGE)	- NAT - All types	1 ESP 1 EST	- Non-operator-dependent technique - SGE is considered an accurate method for apex access.
Ackerman S et al. 2019 [14]	Guide accuracy in OT and AP (with SGE vs. MAN)	- NAT - All types	No data	- The use of 3D guides for endodontic surgery is more accurate.
Leontiev W et al. 2021 [80]	Suitability of IMR instead of CBCT for CA (with SGE)	- NAT - I, C, and PM - maxillae and mandibles	1 operator with 2 years of professional experience	- MRI has an accuracy comparable to CBCT for performing CA.
Janabi A et al. 2021 [30]	Accuracy and efficiency for PR towards pre-treated teeth (with DGE vs. MAN)	- NAT - Maxillary I & C with glass fiber posts	1ESP (with microscope for MAN)	- More accurate and efficient DGE for PR
Perez C et al. 2021 [81]	Reliability for RP when artefacts are present on CBCT(with SGE)	- NAT - PM and M - maxillae and mandibles	2 operators	- SGE is safe and reliable for root canal retreatment, preserving tooth structure. - Can be used by most operators

EG: guided endodontics, VS: versus, DGE: dynamic guided endodontics, SGE: static guided endodontics, MAN: manually, OP: pulp obliteration, AC: cavity opening, NAT: natural, R3D: 3D replicas, ESP: endodontic specialist, EST: student, DG: general dentist, I: incisors, C: canines, PM: premolars, M: molars, OT: osteotomy, AP: apicoectomy, RP: post (fiberglass) removal, CBCT: cone beam computed tomography, and IMR: magnetic resonance imaging.

**Table 2:** Other types of articles included in the review.

Author	Tooth	Diagnosis	Previous Treatment	Trauma	Problem	Type of EG	Results
Todd R et al. 2021 [60]	2.1	NP PAS	No	No	OP	SGE	Tooth without symptomatology after 24 h.
Buchgreitz J et al. 2019 [35]	1.6	PAS	Yes	No	OP	SGE	Tooth without symptomatology after 2 years.
Torres A et al. 2021 [36]	1.4	NP PAS	No	No	OP	SGE	Bone regeneration at one year
Lara Mendes STO et al. 2018 [37]	2.7, 2.8	PAS	No	No	OP	SGE	No symptoms and bone regeneration after one year
Fonseca Tavares WL et al. 2018 [3]	(a) 1.1 (b) 1.1	(a) NP PAS (b) PAS	(a) No (b) Yes	Yes	OP	SGE	(a) At 15 days, there was no symptomatology. (b) Tooth asymptomatic at 30 days.
Lara Mendes STO et al. 2018 [38]	2.1	PAS	No	Yes	OP	SGE	Tooth without symptomatology after 1 year.
Maia LM et al. 2019 [39]	(a) 2.6 (b) 2.5 (c) 1.5	(a) PAS (b) NP Bruxismo (c) PAS	(a) No (b) Yes (c) Yes	No	OP	SGE	Complete healing after 1 year
Fonseca Tavares WL et al. 2020 [40]	2.3	PAS	No	No	OP, complex root anatomy	SGE and photo-dynamic dynamics	Asymptomatic tooth at 12 months.
Fernandes Goncalves W. 2021 [41]	(a) 2.3 (b) 4.6	PAS	(a) No (b) Yes	No	OP RP	SGE	(a) At one year, the size of the apical lesion was reduced and there was no symptomatology. (b) No signs or symptoms at one-year review.
Fonseca Tavares WL et al. 2020 [42]	(a) 4.7 (b) 4.6 (c) 1.6	PAS	(a) Yes (b) Yes (c) Yes	No	OP	SGE	(a) Tooth asymptomatic at 12 months. (b) No data. (c) No symptoms at 12 months.
Maia LM, et al. 2020 [43]	4.6	NP PAS	Yes	No	OP	SGE	Complete healing after 24 months of revision.
Freire BB et al. 2021 [44]	1.1	NP PAS	No	Yes	OP	SGE	Complete healing and absence of symptomatology after 2 years.
Doranala S et al. 2020 [63]	1.1	NP PAS	No	Yes	OP	SGE	Signs of healing at 3 months and absence of symptomatology.
Casadei BDA et al. 2020 [45]	1.5	AAC	Yes	No	OP	SGE	Absence of symptomatology at one year together with a decrease in the size of the apical lesion.
Loureiro MAZ et al. 2021 [9]	2.1	PAA	Yes	Yes	OP	SGE	Satisfactory results at the 6-month checkup.
Villa Machado PA et al. 2022 [46]	3.1	NP PAS	No	Yes	OP	DGE	Asymptomatic at 12 months.
Connert T et al. 2018 [64]	3.1, 4.1	PAS	No	Yes	OP	SGE	There was no symptomatology at 2 weeks.
Torres A et al. 2019 [65]	2.2	PAS	No	No	OP	SGE	Apical lesion healing at 6 months.
Silva AS et al. 2020 [53]	2.1	NP	Yes	No	OP	SGE.	Successful results after 1 year.
Coelho Santiago M et al. 2022 [48]	4.6	NP	Yes	No	OP	SGE	Asymptomatic tooth at one year.
Krug R et al. 2020 [49]	1.5, 1.2, 2.6, 3.6, 3.2, 3.1 and 4.6	PAS Dysplasia dentinaria	Yes, en 3.6	No.	OP	SGE	At one year, there was complete healing of 1.5, 2.6, 3.1, and 4.6, as well as reduction of apical lesion size by 3.6, 3.2, and 1.2.
Kaur G et al. 2021 [50]	2.2	PAS	No	Yes	OP	SGE	Tooth asymptomatic at 2 weeks.
Ali A et al. 2022 [51]	(a) 4.4 (b) 1.1, 1.2, 2.2. (c) 1.2, 2.1	NP PAS	(a) No (b) No (c) No	(a) No (b) Yes (c) Yes	OP	SGE	At one year, there was absence of signs and symptoms in all cases.
Llaquet Pujol M et al. 2021 [52]	(a) 2.1 (b) 1.3 (c) 2.1 (d) 1.1 (e) 1.1 (f) 2.1 (g) 1.1	(a) PAS (b) AAC (c) AAA (d) PAS (e) AAA (f) PAA (g) PAA	No	Yes	OP	SGE	No symptoms at one year.
Yan YQ et al. 2021 [54]	2.7	PAS	Yes	No	OP	SGE (mediate inlay unitario)	No symptoms at two years.
Mena Álvarez J et al. 2017 [55]	2.1	AAC	No	No	Dens evagina-tus	SGE	No symptoms at one year.
Moreira Maia L et al. 2020 [62]	2.1	PAS	Yes	Yes	RT RP	SGE	Injury healing at 18 months.
Perez C et al. 2020 [56]	1.6	PAS	Yes	No	RT RP	SGE	Healing of the periapical area at one year.
Strbac G et al. 2017 [57]	1.5 y 1.6	PAS	Yes	No	OT AP	SGE	Healing of the periapical area at one year.
Giacomino CM et al. 2018 [70]	(a) 1.7 (b) 2.6 (c) 3.5	(a) AAC (b) PAA (c) PAS	(a) Yes (b) No (c) Yes	(a) No (b) Yes (c) No	OT AP	SGE	(a) No symptoms at 12 weeks. (b) Asymptomatic at one month. (c) Asymptomatic at one month.
Popowicz W et al. 2019 [69]	(a) 2.5 (b) 2.5	a) PAS b) NP PAS	(a) Yes (b) Yes	(a) No (b) No	OT AP	SGE	(a) No symptoms at 7 months. (b) No symptoms at 8 months.
Benjamin G et al. 2021 [68]	(a) 2.6 (b) 3.6 (c) 2.6	PAS	YES	No	OT AP	SGE	(a) No symptoms after 10 days. (b,c) No symptoms at 1 week.
Gómez Meda R et al. 2022 [58]	2.3	Impacted	No	No	OP AT AUT	SGE	Complete bone integration at 2 years.
Fu W et al. 2022 [67]	(a) 3.6 (b) 3.6 (c) 2.6	(a) PAS (b) AAA (c) AAA	(a) Yes (b) Yes (c) Yes	No	OT, AP	DGE	(a) Asymptomatic at 9 months. (b) Cure at 6 months. (c) Asymptomatic at 3 months.
Fonseca Tavares WL et al. 2019 [66]	2.5	PAS	Yes	No	OT AP	SGE	Asymptomatic at 6 months.
Chaves GS et al. 2022 [59]	3.6	PAA RRE	Yes	No	OT AP	SGE	Asymptomatic at 1 year.
Gambarini G et al. 2019 [61]	1.2	PAS	Yes	No	OT AP	DGE	Successful healing after 1, 3, and 6 months of control.

NP: pulp necrosis, PAS: symptomatic apical periodontitis, PAA: asymptomatic apical periodontitis, OP: pulp obliteration, AAA: acute apical abscess, AAC: chronic apical abscess, RT: retreatment, RP: removal of post (fiberglass), OT: osteotomy, AP: apicoectomy; ERR: external root resorption, IRR: internal root resorption; AUT: auto-transplantation.

## 4. Discussion

After identifying and describing the available studies, the different applications of guided endodontics would be towards:

**Endodontic access cavities**. Many of the available studies are based on performing an endodontic access cavity, which is the first step in performing non-surgical root canal treatment. Three of the studies [21,22,73] focused on ultra-conservative [21] and minimally invasive [22,73] approaches.

In the study by Gambarini G et al. [21], ultra-conservative access (comparing DGE and manual) consisting of linear access to the teeth was performed with the aim of minimizing tooth weakness, preserving as much tooth tissue as possible, and reducing instrument stress during treatment. Endodontic access cavities are a controversial subject; the terminology is inconsistent [82], and there are multiple classifications for these accesses [47]. They could be broadly classified as: traditional (the pulp chamber roof is removed, and the coronal third of the canals are accessed directly), conservative (the access is made in the central fossa and expanded just enough to locate the canals), ultra-conservative (minimal access in the deepest center of the tooth), and truss access cavities (oval cavities guided by micro-CT imaging where the pulp chamber roof is preserved between the accesses and depending on the diameter of the rotary instruments subsequently used) [83]. However, a linear and direct access in the coronal third would be helpful in reducing the chances of perforations, false passages, or transported canals [21]. Generally, minimally invasive cavities generate a trajectory towards the canal that causes the endodontic instruments to bend and generate stress on the canal. This can lead to iatrogenic accidents such as fractures or steps [83]. However, by means of DGE, this did not occur since the access is direct, linear, and parallel to the axis of the canal [21]. Regarding the loss of dental tissue, it is evident that it is lower in more conservative access cavities than in traditional ones (which is also corroborated in the study). It has been shown that fracture resistance in anterior teeth is not related to the type of endodontic access, in contrast to posterior teeth, where there are certain discrepancies. Some studies show no differences and others state that as long as marginal ridges are preserved, the endodontic access does not negatively influence the tooth’s stiffness [83,84].

The study by Simon GC et al. is an adaptation of the dynamic guided navigation system [73]. In this study, both traditional and minimally invasive, multiple access cavities were performed using CO_2_ laser ablation, providing an alternative method to the use of drills. Using lasers, in this case, means that the infected tissues and toxins are heated to high temperatures, which reduces contamination of the most apical layers of soft or hard tissues. It also produces hemostasis, which may be a treatment option for pulpotomies [73]. However, although the laser used in pulpotomies has a similar clinical and radiographic success rate to other techniques such as MTA and formocresol, it should be taken into account that its use can produce pulp hyperemia due to the heat generated, which can be avoided by removing the affected pulp tissue with manual instruments [85]. Another advantage of its use is that it is possible to perform laser surgery without using CBCT data, since the dynamic navigation system has an integrated digital image of the occlusal surface. This way, the operator can design the access, and it will be performed automatically by the laser, which is controlled by a computer [73].

Concerning other possible applications, starting from the premise that guided endodontics can be applied in difficult cases with calcifications, it is pertinent to think that it could also be used in cases of abnormal dental morphologies that make conventional endodontic treatments difficult. Although there are no studies in this regard, several authors such as Ali A et al. [86], Mena Álvarez J et al. [55], and Zubizarreta Macho A et al. [87] have reported several cases in the literature where 3D splints were used to treat cases of dens invagintus and dens evaginatus with successful results.

**Pulp calcifications**. The most common treatment performed with GE was the treatment of pulp calcifications [3,9,35,36,37,38,39,40,41,42,43,44,45,46,47,48,49,50,51,52,54,58,60,63,64,65]. Jain SD et al. [22] aimed to locate calcified canals by performing minimally invasive cavities using high-speed drills and DGE. It was found that the group that located these canals manually accumulated several errors that resulted in perforations and a greater amount of dental tissue removal. Using high-speed drills entails less operation time compared to low-speed drills used in conjunction with 3D guides in static guided endodontics. This study also showed that using DGE requires some time to learn the technique as manual and visual coordination is required at all times. This makes the results of DGE treatment dependent on the experience of the operator, which is not influenced by DGE, as was also concluded in the study by Connert T et al. [28].

In the study by Connert T et al. [28], as mentioned above, it was confirmed that even an inexperienced operator could have similar success to an endodontic specialist with respect to locating calcified canals, removing a minimal amount of tooth tissue, and completing the treatment in a similar time.

The access cavities made with guided endodontics, especially SGE, are limited to a linear access, which means that they cannot be performed in curved canals or in teeth with an unusual morphology. In the case of straight canals in the same tooth (such as a molars), several guides would have to be designed in the case of DGE (one per root or depending on where the canals are located). A single guide with several accesses could be considered in cases of multiple root canals in adjacent teeth, e.g., several incisors, or a DGE treatment could be planned to perform these root canals in one session [28].

For cases of teeth with OP and canals that are not very curved or with some curvature in the apical third, several management modalities could be combined, such as using SGE or DGE as far as possible and instrumenting the curved part in a conventional way and/or using some treatment such as photodynamic therapy as performed by Fonseca WL et al. [40]. Thus, further studies covering different clinical situations are suggested.

**Osteotomy andapicectomy**. Apicoectomy and osteotomy were the second most common treatment performed with GE [57,59,61,66,67,68,69,70]. A retrospective study by Galino Buniag A et al. [78] is the only one that presents the follow-up of patients who underwent SGE treatment after at least 1 year, showing that it is as valid a treatment option as the conventional one (performing a full-thickness flap and using drills and reamers). However, it does not report the process of the SGE performed. More studies similar to this one with longer follow-ups of patients treated with both types of guided endodontics are needed.

In the studies by Ackerman S et al. [14] and Fan Y et al. [15], direct access to the apex was made by drilling through the bone. In the study by Ackerman S et al. [14], a flap was also made (as well as in the studies by Aldamash SA et al. [29] and Gaffuri S et al. [79]) to simulate the clinical conditions of endodontic microsurgery. In other studies, however, such as that of Smith GB et al. [77], it was proposed to forego performing a flap and to use a biopsy at the site where the osteotomy would be performed to remove the masticatory palatal mucosa (which will then be sutured after the completion of treatment). This represents a new approach to minimally invasive endodontic microsurgery with the advantage of being more comfortable for the patient in the postoperative process and avoiding damage to compromised structures such as the greater palatal artery in the case of apicoectomies of palatal roots of maxillary molars. This is reported by authors such as Shcmid C et al. [88], Benjamin G et al. [68], and Giacomino CM et al. [70].

**Glass fiberposts’ removal**. The re-treatment of teeth that require fiberglass posts’ removal was the third most common treatment performed with GE [43,56]. The studies by Perez C et al. [81] and Janabi A et al. [30] cover this topic using SGE and DGE, respectively. The removal of fiberglass posts is mainly carried out after a previously failed treatment [81] and it can be performed with the help of ultrasound tips [89]. Still, it involves a risk of the perforation of the tooth [30]. In addition, the color of the post, which blends in with the adjacent dentine, is an added difficulty [90]. Even so, the practitioner’s experience performing this treatment influences the amount of extra dentine removed around the post, which is greater and leads to a widening of the radicular canal after the removal of the post [91]. For these reasons, guided endodontics is suggested as a treatment alternative.

In the study by Perez C et al. [81], which was carried out with SGE, the apical gutta-percha could be accessed in 87.5% of the treated teeth, and those that could not be fully accessed were due to root curvature. Furthermore, this study is interesting because it simulates artifacts in the CBCT images, which makes access design and guidance difficult, as they are not as accurate. Even so, the results were satisfactory and require less time than ultrasonic tips or long-stem drills. Once again, it was confirmed that this procedure could be performed by any operator.

Similarly, the study by Janabi A et al. [30] also had satisfactory results and faster results compared to removing the post by milling and using a microscope. Still, the operator needed to adapt to this new system to work comfortably and quickly.

The limitations of the present work are based on the type of studies covered in the literature since guided endodontics is a new topic that is only beginning to be developed, expanded, and applied. Therefore, more and higher quality studies are needed in the future, such as randomized clinical trials, to compare the results of all—and other future—applications of SGE and DGE in real patients and with long-term clinical and radiological follow-ups. Even so, one of the risks of bias presented by the selected articles is that not all the procedures were performed with extracted teeth; some were 3D-printed, in which case the tooth’s characteristics are not the same as in a real tooth. In addition, in other types of articles, the follow-up periods were short, some even of days or weeks, which is not enough time to evaluate the outcome of the use of the technique.

## 5. Conclusions

EG applications encompass not only endodontic cavity access and canal location with PO but can also be applied in cases of osteotomy and apicoectomy as well as retrograde fillings, the removal of fiberglass posts, and treating teeth with morphological asymmetries.

- The advantages of SGE are as follows: it is independent of the operator’s experience, requires less treatment time for the patient, and is more accurate and safer than conventional endodontics.
- The disadvantages of SGE are as follows: more time is needed for the design and production of 3D guides, it involves linear access that only works for straight canals, and it is not very stable in the mouth in partially edentulous patients.
- The benefits of DGE are as follows: it is more ergonomic (in terms of having to look at the monitor during treatment), it allows for the real-time adjustment and repositioning of the working instruments, it is more accurate as it does not accumulate design errors, and it is useful in cases of multi-rooted teeth.
- The disadvantages of DGE are as follows: it is highly dependent on the operator’s experience and requires deeper learning for its mastery, and it requires simultaneous hand–eye coordination.

## Data Availability

Not applicable.
